# Occurrence of the Elongated Styloid Process on Digital Panoramic Radiographs in the Riyadh Population

**DOI:** 10.1155/2021/6097795

**Published:** 2021-11-11

**Authors:** Lingam Amara Swapna, Nada Tarek AlMegbil, Alhanouf Othman Almutlaq, Pradeep Koppolu

**Affiliations:** ^1^Department of Surgical and Diagnostic Sciences, College of Dentistry, Dar Al Uloom University, Riyadh 13314, Saudi Arabia; ^2^Department of Preventive Dental Sciences, College of Dentistry, Dar Al Uloom University, Riyadh 13314, Saudi Arabia

## Abstract

**Background:**

Patients with an elongated styloid process might present with dysphagia and pain in the cervicofacial region. These patients could be misdiagnosed as other orofacial pathologies.

**Aim:**

The present study attempted to assess the prevalence of the elongated styloid process on digital panoramic radiographs in the Riyadh population.

**Materials and Methods:**

The present prospective randomized study was conducted on the panoramic digital radiographs of 300 randomly selected patients visiting a private dental hospital to identify any elongation of the styloid process. Only the radiographs without any magnification errors were considered. The styloid process length was measured using the Sidexis measuring tool and entered in an Excel spreadsheet with other demographic data. A length beyond 30 mm was considered styloid process elongation. The data were subjected to statistical analysis.

**Results:**

The symptoms of styloid process elongation were higher among females (78.6%), and this difference was statistically significant (*χ*^2^ = 7.182; *P*=0.007). No statistically significant association was observed between styloid process elongation and symptoms between different age groups. Females exhibited a significant longer mean length of the styloid process than males. The present study exhibited a 27.3% prevalence for the elongation and calcification of the styloid process.

**Conclusion:**

Given the significant prevalence of the elongated styloid process in our study, we recommend it to be considered as one of the differential diagnosis for pain or discomfort in the orofacial region.

## 1. Introduction

The styloid process is a slender bony process starting from the lower portion of the temporal bone anteromedial to the stylomastoid foramen [1]. The term styloid has originated from the Greek word “stylos,” which means “pillar” [2]. The process is attached to the lesser cornu of the hyoid bone through the styloid ligament and is flanked on both sides by the external and internal carotid arteries [3]. The normal length of the styloid process is from 15 mm to 30 mm. A length of >30 mm is regarded as elongated [4]. The prevalence of an elongated styloid process in the general population ranges 2%–52% in various studies [5, 6].

The symptomatic elongation of the styloid process is termed as Eagle syndrome. This pathology was first reported in 1937 by the otorhinolaryngologist, Eagle, who termed it stylalgia [7]. Patients with Eagle syndrome present with dysphagia, pain in the cervicofacial region and neck, and pain at the mandibular angle, which aggravate with neck rotation or tongue protrusion. These features may be due to the pressure that the elongated styloid process exerts on the adjacent neurovascular structures [8]. However, the presentation of this pathology may be confusing in a clinical setting and may be misinterpreted as unerupted or impacted tooth, cranial nerve neuralgias, temporal arthritis, temporomandibular joint disorders, migraine, pharyngotonsillitis, and benign and malignant tumors [9, 10]. This may lead to unnecessary invasive diagnostic and therapeutic procedures. Additionally, an elongated styloid process may also cause stroke due to the compression of carotid arteries [11].

Thus, an elongated styloid process must be diagnosed accurately and considered as a differential diagnosis in orofacial pain. Therefore, the present study attempts to evaluate the prevalence of styloid process elongation and its association with demographic factors in the population in Riyadh.

## 2. Materials and Methods

The present cross-sectional observational study was conducted in 300 patients above 14 years of age in Dar Al Uloom University Hospital, Riyadh, Saudi Arabia, from August 2020 to April 2021, after institutional ethics clearance. The study was conducted according to the guidelines of the Declaration of Helsinki and approved by the Institutional Review Board of College of Dentistry, Dar Al Uloom University, Riyadh, KSA (COD/IRB/2020/5). Both verbal and written consent was obtained from all patients. The patients visiting the dental clinics were randomly selected. Radiographs clearly exhibiting bilateral styloid processes with minimal or no magnification were included in the study. Radiographs exhibiting magnification errors were excluded from the study.

The digital panoramic radiographs of all 300 patients were obtained, and data regarding the same were obtained from the university hospital systems (Sidexis). These panoramic radiographs were taken with a digital panoramic orthophos XG system (Sirona Company) using exposure factors as per manufacturer' instruction. The length of the styloid process was measured from the junction of the process and the tympanic plate to the tip of the process. In case of a segmented styloid process, the measurement was performed in the same manner. The ossification of the stylohyoid or stylomandibular ligaments, if present, was considered a part of the elongated styloid process, and measurements were performed accordingly. A styloid process greater than 30 mm long was considered as elongated.

**Figure 1:**
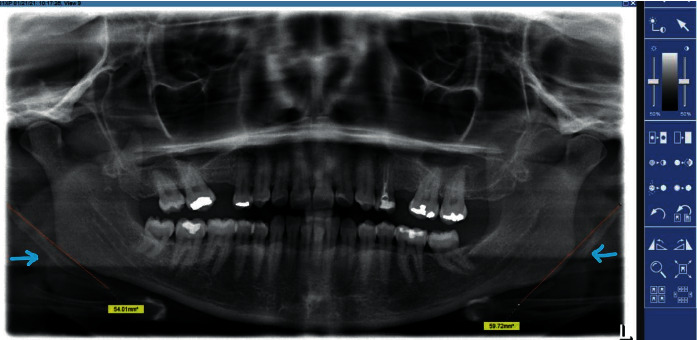
Measuring the elongated styloid process on both sides.

The patients were interviewed in either Arabic or English depending on their comfort. Extraoral examination was then carried out. The neck region of the patients was palpated for any signs of styloid process elongation. Additional patient information was obtained from the hospital electronic file system (Open Dental). A Sidexis ruler was used to calculate the length of the styloid process in the radiographs (Figure 1).

The data were collected and entered in MS Excel. The results were analyzed using SPSS version 22.0. The association between the different parameters was analyzed using the chi-square test, and *P* < 0.05 was considered statistically significant.

## 3. Results

The characteristics of the study population are given in Table 1. In the present study, majority of the samples were males (*n* = 166; 55.3%). The majority of patients were in the age group of 20–29 years (34%), followed by 30–39 years (23.3%), 40–49 years (19.3%), above 50 years (12.7%), and less than 20 years (10.7%). The prevalence of elongation and calcification patterns of the styloid process was 27.3% (*n* = 82). Among the patients with positive symptoms (*n* = 28; 9.3%), 4.7% (*n* = 14) exhibited symptoms on the right side and 6% (*n* = 18) exhibited symptoms on the left side. Of these, 4 patients had bilateral symptoms.

Chi-square analysis displayed no statistically significant association among styloid process elongation (*χ*^2^ = 4.909; *P*=0.297), symptoms (*χ*^2^ = 3.357; *P*=0.5), and sides of the symptoms (*χ*^2^ = 3.78; *P*=0.706) with different age groups. Chi-square analysis displayed significantly higher symptoms of styloid process elongation in females (78.6%) compared with males (21.4%) (*χ*^2^ = 7.182; *P*=0.007). No significant difference was observed in the occurrence of styloid process elongation and symptoms on left and right between males and females (*P* > 0.05).

Table 2 provides the association of styloid process elongation, symptoms, and sides of the symptoms between different age groups. No statistically significant association was observed between styloid process elongation (*χ*^2^ = 4.909; *P*=0.297), symptoms (*χ*^2^ = 3.357; *P*=0.5), and sides of the symptoms (*χ*^2^ = 3.78; *P*=0.706) between different age groups.

Table 3 provides the association of styloid process elongation, symptoms, and sides of the symptoms between males and females. No statistically significant difference was observed between styloid process elongation between males and females (*P* > 0.05). However, the symptoms of styloid process elongation were higher among females (78.6%), and this difference was statistically significant (*χ*^2^ = 7.182; *P*=0.007).

## 4. Discussion

Eagle syndrome may cause symptoms such as orofacial pain, which might be confusing to clinicians [8]. All clinicians must be aware of the clinical and radiological signs and symptoms of styloid process elongation to accurately diagnose and treat symptoms in the cervicofacial region. The present study thus attempts to evaluate the prevalence of styloid process elongation in the Riyadh population and its association with demographic factors.

The present study was conducted on digital panoramic radiographs. Numerous imaging modalities can be used for the diagnosis of styloid process elongation. These include modalities such as lateral skull radiograph, Towne's view, anterioposterior skull radiograph, and computed tomography (CT) scan [12]. However, digital panoramic radiographs can be easily performed and interpreted. Additionally, they expose the patients to a lower radiation dose and cost much lesser than CT. Thus, this procedure can be easily used for epidemiological evaluations [13]. Furthermore, digital panoramic radiographs are commonly prescribed for orofacial pain, and a styloid process elongation is usually an accidental finding on these radiographs. So, we opted for this diagnostic modality in the present study.

The present study exhibited a prevalence of 27.3% for the elongation and calcification of the styloid process. This finding was concurrent with those of Bozkir et al., Keur et al., and Scaf et al., who exhibit prevalence rates less than 30% [14]. However, this finding was in contrast with the findings of AlZarea and Bagga et al. [2, 6]. Bagga et al. reported a prevalence of 52.1% for the elongated styloid process. This difference in prevalence may be explained by the differing geographic area as the study was conducted in Mathura. The authors explained this high prevalence by the fact that majority of the population perform strenuous works such as carrying heavy weights on their heads and chew hard foods such as gutka and areca nut, which may promote the ossification of the ligaments attached to the styloid process [6]. However, the study on AlZ area, exhibiting a prevalence of 43.93% for the elongated styloid process, was conducted in Saudi Arabia itself. However, the population studied was >60 years of age. As the present study included patients as young as 14 years, the age difference in the study population may account for the difference in prevalence.

The difference in the length of the styloid process between different age groups in this study was statistically nonsignificant. This finding was in contrast to the findings of More and Asrani, Bruno et al., and Jamal et al., who reported a progressive increase in the length of the styloid process with aging [12–15]. Additionally, the present study presented a longer mean length of the styloid process in females (right side: 16.04 ± 16.88 mm; left side: 16.44 ± 17.31 mm) compared with males (right side: 12.51 ± 16.38 mm; left side: 13.86 ± 16.89 mm). This finding is in contrast with those of More and Asrani and Jamal et al., who reported longer styloid processes in males as compared with females [12, 15]. However, these findings were concurrent with the findings of Magat and Ozcan, who reported no significant difference in the length of the styloid process between males and females, and Ferrario et al., who reported a longer styloid process length in females [16, 17].

Out of the 34 females with elongated styloid processes in the present study, 22 (78.60%) exhibited symptoms, whereas only 6 (21.40%) of the 48 males with elongated processes exhibited symptoms. This difference was statistically significant (*P*=0.007). This may be due to the lower pain threshold in females [18].

The present study has certain limitations. Langlais et al. classified the radiographic images of the elongated styloid process into three types based on morphology of elongation, namely, type I: elongated, type II: pseudoarticulated, and type III: segmented [19]. The present study did not take this classification under consideration. The relatively small sample size and the single-centre design of the study prevent the generalization of the findings. Additionally, digital panoramic radiography is prone to magnification errors, which may confound findings. Future multicentre studies with a larger sample size will further strengthen the findings of this study.

## 5. Conclusion

As styloid process elongation may be an accidental finding during radiographic examination, practitioners must remember to look for the same when patients visit with pain in the cervicofacial region. The present study exhibited a prevalence of 27.3% for the elongation and calcification of the styloid process. Thus, it must be considered as a differential diagnosis for pain or discomfort in the orofacial region.

## Figures and Tables

**Table 1:** Characteristics of the study population.

Age and sex distribution in the population				
			Frequency	%
Age groups	Less than 20 years		32	10.7
	20–29		102	34
	30–39		70	23.3
	40–49		58	19.3
	Above 50 years		38	12.7
Sex	Female		134	44.7
	Male		166	55.3

Mean age among sexes				
		*N*	Mean age	SD
Sex	Female	134	31.49	11.70
	Male	166	35.47	13.67
Total		300	33.69	12.94

Distribution of elongation of the styloid process				
			Frequency	%
Elongated	No		218	72.7
	Yes		82	27.3

Distribution of symptoms between sides				
			Frequency	%
Symptoms	No		172	90.7
	Yes		28	9.3
Symptoms right	No		286	95.3
	Yes		14	4.7
Symptoms left	No		282	94
	Yes		18	6

*N*, number of samples; SD, standard deviation.

**Table 2:** Association of styloid process elongation, symptoms, and sides of the symptoms between different age groups.

Mean length among age groups (in mm)										
Age groups							Right		Left	
Less than 20 years		*N*					32		32	
		Mean					13.40		12.73	
		SD					17.03		15.03	
20–29		*N*					102		102	
		Mean					12.85		11.43	
		SD					17.01		16.93	
30–39		*N*					70		70	
		Mean					16.22		19.81	
		SD					17.56		18.07	
40–49		*N*					58		58	
		Mean					13.77		16.69	
		SD					15.89		17.14	
Above 50 years		*N*					38		38	
		Mean					14.54		15.15	
		SD					16.06		16.23	
Total		*N*					300		300	
		Mean					14.09		15.01	
		SD					16.64		17.07	

Association of styloid process elongation among different age groups										
			Age groups					*χ* ^2^ value		*P* value
			Less than 20 years	20–29	30–39	40–49	Above 50 years			
Elongated	No	*N*	28	70	44	46	30	4.909		0.297
	Yes	*N*	4	32	26	12	8			
Symptoms	No	*N*	30	88	64	52	38	3.357		0.5
	Yes	*N*	2	14	6	6	0			

^*∗*^Statistical significance set at 0.05; *N*, number of samples; *χ*^2^ value, chi-square value.

**Table 3:** Association of styloid process elongation, symptoms, and sides of the symptoms between males and females.

Mean length among males and females (in mm)						
Sex					Right	Left
Female		*N*			134	134
		Mean			16.04	16.44
		SD			16.88	17.31
Male		*N*			166	166
		Mean			12.51	13.86
		SD			16.38	16.89
Total		*N*			300	300
		Mean			14.09	15.01
		SD			16.64	17.07

Association of styloid process elongation among males and females						
			Sex		*χ* ^2^ value	*P* value
			Female	Male		
Elongated	No	N	100	118	0.234	0.628
	Yes	N	34	48		
Symptoms	No	N	112	160	7.182	0.00^7*∗*^
	Yes	N	22	6		

^*∗*^Statistical significance set at 0.05; *N*, number of samples; *χ*^2^ value, chi-square value.

## Data Availability

The data used to support the findings of this study are available from the corresponding author upon request.
